# In Search for the Missing Link in APECED-like Conditions: Analysis of the *AIRE* Gene in a Series of 48 Patients

**DOI:** 10.3390/jcm11113242

**Published:** 2022-06-06

**Authors:** Alessandra Fierabracci, Eugenia Belcastro, Elena Carbone, Olivia Pagliarosi, Alessia Palma, Lucia Pacillo, Carmela Giancotta, Paola Zangari, Andrea Finocchi, Caterina Cancrini, Domenico Vittorio Delfino, Marco Cappa, Corrado Betterle

**Affiliations:** 1 Infectivology and Clinical Trials Research Department, Bambino Gesù Children’s Hospital, Scientific Institute for Research, Hospitalization and Healthcare (IRCCS), 00146 Rome, Italy; eugenia.belcastro@opbg.net (E.B.); elena.carbone@opbg.net (E.C.); olivia.pagliarosi@opbg.net (O.P.); 2 Research Laboratories, Bambino Gesù Children’s Hospital, IRCCS, 00146 Rome, Italy; alessia.palma@opbg.net; 3 Academic Department of Pediatrics (DPUO), Immune and Infectious Diseases Division, Research Unit of Primary Immunodeficiencies, Bambino Gesù Children’s Hospital, IRCCS, 00165 Rome, Italy; lucia.pacillo@opbg.net (L.P.); andrea.finocchi@opbg.net (A.F.); caterina.cancrini@opbg.net (C.C.); 4 PhD Program in Immunology, Molecular Medicine and Applied Biotechnology, University of Rome “Tor Vergata”, 00133 Rome, Italy; 5 Immunology and Vaccinology, DPUO, Bambino Gesù Children’s Hospital, IRCCS, 00165 Rome, Italy; carmela.giancotta@opbg.net (C.G.); paola.zangari@opbg.net (P.Z.); 6 Chair of Pediatrics, Department of Systems Medicine, University of Rome “Tor Vergata”, 00133 Rome, Italy; 7 Department of Medicine and Surgery, University of Perugia, 06123 Perugia, Italy; domenico.delfino@unipg.it; 8 Endocrinology Unit, DPUO, Bambino Gesù Children’s Hospital, IRCCS, 00165 Rome, Italy; marco.cappa@opbg.net; 9 Endocrine Unit, Department of Medicine (DIMED), University of Padua, 35128 Padua, Italy; corrado.betterle@unipd.it

**Keywords:** autoimmunity, immunodeficiency, APECED-like conditions, *AIRE* gene polymorphisms, autoantibodies, precision medicine, candidate gene approach, whole-exome sequencing, diagnostic workup, targeted therapies

## Abstract

Autoimmune diseases are a heterogeneous group of disorders of the immune system. They can cluster in the same individual, revealing various preferential associations for polyendocrine autoimmune syndromes. Clinical observation, together with advances in genetics and the understanding of pathophysiological processes, has further highlighted that autoimmunity can be associated with immunodeficiency; autoimmunity may even be the first primary immunodeficiency manifestation. Analysis of susceptibility genes for the development of these complex phenotypes is a fundamental issue. In this manuscript, we revised the clinical and immunologic features and the presence of *AIRE* gene variations in a cohort of 48 patients affected by high polyautoimmunity complexity, i.e., APECED-like conditions, also including patients affected by primary immunodeficiency. Our results evidenced a significant association of the S278R polymorphism of the *AIRE* gene with APECED-like conditions, including both patients affected by autoimmunity and immunodeficiency and patients with polyautoimmunity compared to healthy controls. A trend of association was also observed with the IVS9+6 G>A polymorphism. The results of this genetic analysis emphasize the need to look for additional genetic determinants playing in concert with *AIRE* polymorphisms. This will help to improve the diagnostic workup and ensure a precision medicine approach to targeted therapies in APECED-like patients.

## 1. Introduction

Autoimmune diseases are a heterogeneous group of disorders of the immune system. Environmental factors, family history and/or genetic susceptibility underlie their etiopathogenesis [1]. These disorders are due to a loss of tolerance to self-proteins or autoantigens that can be organ specific or systemic [2]. Organ-specific autoimmune diseases are due to target cell destruction determined by autoreactive T lymphocytes and can cluster in the same individual revealing various preferential associations; this is the case of polyendocrine autoimmune syndrome Type I (autoimmune polyendocrinopathy candidiasis ectodermal dystrophy syndrome (APECED)), Type II and immune dysregulation, polyendocrinopathy, enteropathy, X-linked (IPEX) syndrome [3]. Indeed, although phenotypically different and confirmed by different diagnostic procedures, autoimmune disorders can share similar immune and genetic defects, a phenomenon called ‘autoimmune tautology’ [2], i.e., the co-occurrence of polyautoimmunity or multiple autoimmune syndrome (MAS) and familiarity for autoimmunity [4]. 

Clinical observation has further highlighted that autoimmunity can even share some common characteristics and mechanisms with other conditions that initially were considered independent polar opposites. Indeed, this was suggested by the high prevalence of autoimmune manifestations in primary immunodeficiencies (PID) and the observation that autoimmunity may even be the first manifestation [5]. 

Identifying susceptibility genes for these complex phenotypes and unraveling their putative effects in their etiopathogenesis is a relevant issue. Further increased awareness and use of genetic screening of confirmatory functional studies, together with immunological markers, can lead to a precision medicine workup for early specific diagnosis in highly vulnerable patient categories [6].

Both purely autoimmune conditions and PIDs can exhibit defects in central and peripheral tolerance influenced by mutations in genes that regulate immunological tolerance [5]. In addition to human leukocyte antigen (HLA) haplotypes [7], several single-nucleotide polymorphisms (SNPs) were discovered to underlie the pathogenesis of autoimmune phenotypes [8]. Examples of common susceptibility genes involved in immune regulation include cytotoxic T lymphocyte-associated antigen 4 (CTLA4), which suppresses T-cell activation [9,10,11], forkhead box P3 (FOXp3), involved in the differentiation of T regulatory cells (Tregs) [12,13], and the interleukin-2 receptor (IL-2R)α/CD25 gene, which affects the development and function of Tregs [14]. Further, polymorphisms of the tumor necrosis factor (TNF)-α gene, located on chromosome 6p21.3, increase the risk of association of insulin-dependent diabetes mellitus (Type 1 diabetes, T1D) and autoimmune thyroid disease [15] and the association of alopecia areata and vitiligo [16]. Among the others, the C1858T polymorphism of the protein tyrosine phosphatase non-receptor type 22 (*PTPN22)* gene is associated with several autoimmune diseases; this encodes for a more active phosphatase, namely the Lyp variant R620W, which is a potent inhibitor of T-cell activation [17,18].

Regarding complex autoimmunity phenotypes, the APECED syndrome (OMIM#240300) [19] is a rare autosomal recessive disease caused by mutations in the autoimmune regulator (*AIRE*) gene [20,21]. The encoded AIRE protein is a transcription factor with an important role in regulating the escape of autoreactive T cells from the thymus in perinatal age and the development of Tregs [19,22]. Classic diagnostic criteria for APECED is the presence of two of the following manifestations: chronic mucocutaneous candidiasis (CMC), chronic hypoparathyroidism (CHP) and Addison’s disease (AD) [23]. Indeed, CMC is often the first clinical manifestation in APECED patients in which multiple organ- and non-organ-specific autoimmune conditions may subsequently develop during their lifetime [23]. Anti-interferon omega (IFNω) antibodies circulating at high titers are serological hallmarks of the syndrome [24]. 

The presentation of self-antigens in the thymus that might favor the development of certain organ-specific autoimmune disorders is also conceived to be influenced by genetic variability in the *AIRE* locus and the presence of heterozygous loss-of-function mutations of the *AIRE* gene [25,26]. In this regard, *AIRE* variants have indeed already been reported in the DNA of patients affected by organ-specific autoimmune disorders [27,28,29,30,31,32,33,34,35,36,37,38,39,40,41,42,43,44,45,46]. Of note in parents of APECED patients harboring heterozygous *AIRE* mutations, immunological dysregulation was detected in the peripheral blood by elevated levels of IgA and activated T lymphocytes [28]. Furthermore, *AIRE* gene monoallelic mutations located in the first plant homeodomain (PHD1) zinc finger with autosomal dominant inheritance were found associated with autoimmune diseases characterized by a later onset, milder phenotype and reduced penetrance; however, manifestations in these conditions did not satisfy the clinical diagnostic criteria for APECED [47]. A milder phenotype was reported in a ’non-classical late onset’ APECED due to a dominant-negative monoallelic mutation (G228W) located in the SAND domain of the *AIRE* gene in an Italian family with high incidence of Hashimoto’s thyroiditis (HT) [48]. Instead, heterozygous recessive *AIRE* gene mutations may, although minimally, contribute to the occurrence of sporadic non-mendelian autoimmunity in the general population [49]. Of note, genome-wide association studies (GWAS) conducted in European cohorts of patients affected by pernicious anemia revealed rs74203920 missense variant leading to R471C substitution (p.Arg471Cys) in the second PHD (PHD2) of the *AIRE* gene among the identified risk loci [50]. Two independent signals rs74203920 and the intronic rs2075876 of the *AIRE* gene were also detected as significantly associated with Addison’s disease in the Swedish population. The last SNP was in linkage disequilibrium with SNP rs1800520 coding for the S278R variant [51].

In a preliminary investigation, we demonstrated the trend of increased association of *AIRE* gene variants, particularly the S278R polymorphism, in patients affected by autoimmune polyendocrinopathies than in healthy controls [46]. The association of the S278R *AIRE* polymorphism was also reported with other autoimmune conditions, including hepatitis, alopecia areata, systemic sclerosis associated with HT and sporadic AD [34,35,36,37,40,41,42]. 

In light of the foregoing, the present study aimed to analyze the *AIRE* gene in a different group of patients affected by even higher polyautoimmunity complexity compared to the previously published cohort [46]. The present screened APECED-like population included variable associations of endocrine and non-endocrine and even immune-dysregulatory conditions manifested as immunodeficiency symptoms/confirmed PIDs and allergies. We also estimated the frequency of the detected *AIRE* gene variants and discussed their putative involvement in the pathophysiological process leading to their clinical and immunological features.

## 2. Materials and Methods

### 2.1. Subjects

A total of 48 patients affected by APECED-like disease, including variable association of organ- and non-organ-specific autoimmune disorders and immunodeficiency-associated conditions (16 males, 32 females with age ranges at presentation between 1 and 15.42 years), were recruited from the University Department of Pediatrics (DPUO), at Bambino Gesù Children’s Hospital (OPBG) in Rome. The patients’ sera were assayed for insulin-dependent diabetes mellitus (Type 1 diabetes (T1D))-related autoantibodies (Abs), i.e., glutamic acid decarboxylase (GADA) (isoform 65), tyrosine phosphatase-related islet antigen 2 (IA2) and insulin (IAA) Abs, for anti-adrenal Abs by radioimmunoassay (RIA), for thyroid-related Abs, i.e., TSH-receptor Abs (TRAb immunoassay, Immulite TSI, Siemens Healthcare, Tarrytown, NY, USA), thyroglobulin (Tg), and thyroperoxidase (TPO) and for celiac-disease-related transglutaminase (TRG) Abs by chemiluminescence (ADVIA Centaur analyzer, Siemens Healthcare, Erlangen, Germany), gliadin, extractable nuclear antigen (ENA), endomysial (EMA) Abs, anti-liver kidney microsomal (LKM) and parietal cells Abs (APCA) by indirect immunofluorescence (IFL). Non-organ-specific Abs anti-nuclear (ANA), anti-neutrophil cytoplasmic (ANCA), anti-double-stranded DNA (dsDNA), anti-reticulin (ARA), anti-mitochondrial (AMA) and anti-smooth muscle cell (ASMA) were also tested. IFNω Abs were assayed by RIA in collaboration with FIRS Laboratories RSR Ltd. (Cardiff, UK). Informed consent was obtained from all those who took part in the present study in accordance with the Declaration of Helsinki. The investigation was approved by the local Institutional Review Board (IRB) of Bambino Gesù Children’s Hospital (OPBG), which regulates human sample usage for experimental studies (Study Protocol No.: 1385_OPBG_2017). A control group included 84 healthy blood donors (44 females and 40 males) [46]. Controls were recruited from the OPBG Blood Transfusion Centre; they had no history of autoimmunity and immunodeficiency and no autoantibodies were detected in their serum. 

### 2.2. Molecular Studies

Genomic leukocyte DNA was extracted from whole blood samples of patients by the QIAmp DNA Blood Mini Kit (Qiagen, Hilden, Germany) according to the manufacturer’s guidelines.

#### AIRE Gene Screening

All 14 exons and intronic regions of the *AIRE* gene were sequenced according to already described protocols (Genetic Analyzer 3500 Applied Biosystems HITACHI system, Thermo Fisher Scientific, Rodano, Italy) in the DNA of recruited patients [46].

### 2.3. Statistical Analysis

Differences in the number of subjects with S278R polymorphism or the IVS9+6 G>A intronic variation of the *AIRE* gene between patients and healthy controls were assessed by the χ2 (chi-square) test on variances and the GraphPad Prism Software (version 7, San Diego, CA, USA). A value of *p* < 0.05 was considered significant.

## 3. Results

Clinical Phenotype and AIRE Gene Screening in APECED-Like Patients

**Figure 1:**
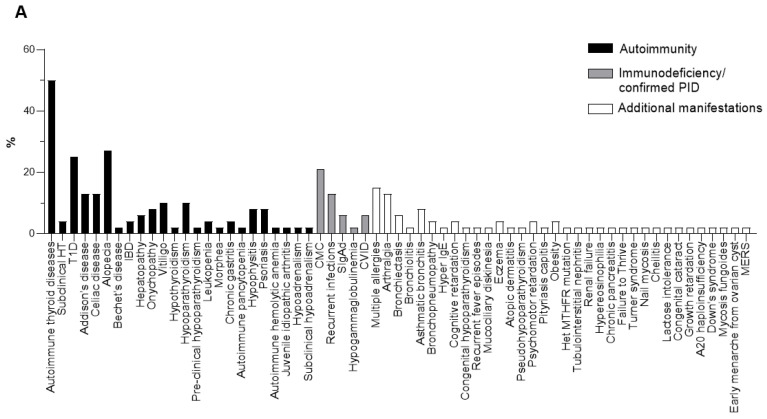

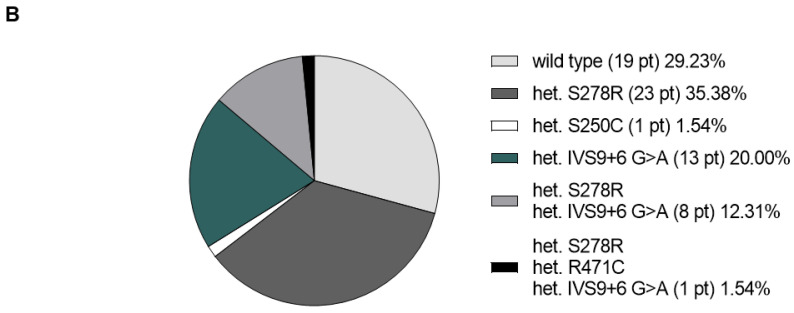
(**A**) Prevalence (%) of clinical manifestations and (**B**) prevalence of S278R, IVS9+6 G>A polymorphism, R471C mutation and S250C variant in the APECED-like patients.

The 48 APECED-like patients of the present series (Table 1) presented variable combinations of autoimmune manifestations both organ and non-organ specific, with a higher prevalence of T1D and autoimmune thyroid diseases, alopecia, vitiligo and Addison’s disease among the others (Figure 1A). Of note, Addison’s disease is one of the major symptoms of APECED, while T1D occurs as a rare manifestation of this syndrome [52]. Out of the total 48 patients, 15 patients (Patients 1, 2, 3, 4, 5, 6, 7, 8, 9, 10, 12, 14, 16, 19 and 20) presented clinical manifestations traditionally associated with PID, including mucocutaneous candidiasis (n = 10) and recurrent infections (n = 6), and 7 of them were actually diagnosed with PID: combined immunodeficiency (n = 2, Patients 5 and 6), common variable immunodeficiency (n = 1, Patient 20) and selective IgA deficiency (SIgAd) (n = 4, Patients 3, 4, 8 and 12). Moreover, seven patients manifested allergies; one had hyper IgE, seven atopic dermatitis and two eczematous dermatitis. Of note, SIgAd patients, as already reported, presented peculiar autoimmune conditions, i.e., T1D (Patient 3), autoimmune thyroiditis (Patients 4 and 8) and vitiligo (Patients 4 and 8) and allergies (Patients 3 and 8) (Table 1) [53]. Patients with autoimmunity and immunodeficiency (Table 1) were tested for anti-IFNω Abs; these specificities are detected in over 90% of APECED patients [20]. The serum of one patient (Patient 11 of the present series) with polyallergy and hypereosinophilia was positive for anti-IFNω Abs. Of note, anti-IFNω Abs were also found positive in the serum of Patient 34 affected by central hypoadrenalism, HT, bronchiectasis and chronic inflammatory demyelinating polyneuropathy (CIDP).

In this APECED-like series of patients, the heterozygous S278R (c.834 C>G, p.Ser278Arg, G961C, rs1800250, exon 7) polymorphism of the *AIRE* gene was detected in 23 out of 48 individuals (Table 1, Figure 1B). The intronic polymorphism IVS9+6 G>A (c.1095+6 G>A, G11107A, rs1800525, intron 9), previously reported in patients affected by autoimmune conditions, including in our own investigation on polyendocrinopathies [46], was detected in 13 patients of the present series. The heterozygous compound S278R/IVS9+6 G>A was reported in eight patients (Table 1). One patient (Patient 3, Table 1) presented in compound heterozygosity S278R/R471C (c.1411 C>T)/IVS9+6 G>A and one patient (Patient 20, Table 1) the S250C variant (c.748 A>T, p.Ser250Cys, rs141480813, exon 6) [54] (Figure 1B).

**Figure 2:**
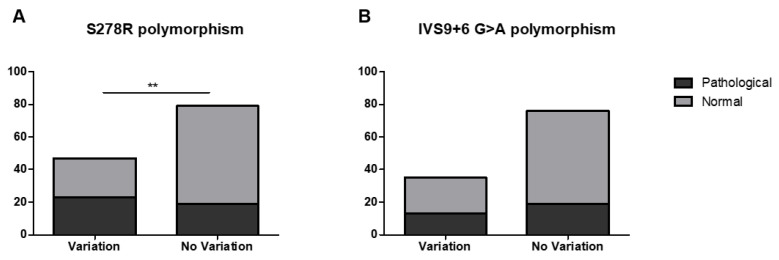
Statistical analysis (χ2 test) of differences in the prevalence of *AIRE* gene polymorphism in patients with APECED-like versus controls. (**A**) Prevalence of the S278R polymorphism in n = 23 patients out of the total n = 48 and controls. Statistically significant difference was observed, ** *p* < 0.05. (**B**) Prevalence of the IVS9+6 G>A intronic polymorphism in n = 13 patients of the total n = 48 versus controls. No statistically significant difference was observed.

The difference in prevalence of the S278R polymorphism between the patient group and the healthy controls was statistically significant (Figure 2A). A trend of increase in the prevalence of the IVS9+6 G>A polymorphism between the patient group and the healthy controls was observed (Figure 2B). These data suggest the putative influence of *AIRE* gene polymorphisms in APECED-like conditions, which is particularly evident in Patient 3 (Table 1), where polymorphism S278R is present in compound heterozygosity with the known R471C *AIRE* mutation [30].

## 4. Discussion

Clinical examination of the series of patients has helped to raise physicians’ awareness of the possible development of different autoimmune manifestations at different ages in the same individual; autoimmunity may even be the first manifestation of PID [2]. Nowadays, PIDs include more than 430 entities [55,56], and they are associated with polyautoimmunity; this is especially applied to CVID and CID. 

In light of the foregoing, preventing the development of polyautoimmunity is a fundamental task [6]. Furthermore, identifying a PID in a heterogeneous group of patients with several autoimmune disorders can also be a difficult task. In the presence of polyautoimmunity, immunologic evaluation should be included at the initial diagnostic workup in order to avoid significant delay of a specific diagnosis in vulnerable patients affected by genetic immune defects. 

In previous studies, a high frequency of certain polymorphisms of the *AIRE* gene, including S278R, were discovered in autoimmune patients including those with non-APECED autoimmunity [37,46] (vide supra). 

In order to validate the influence of susceptibility genes in the pathogenesis of complex autoimmune phenotypes, in the present investigation, we searched for *AIRE* gene variants in a population of APECED-like patients. Seven patients were also diagnosed with PID, and in some of them, recurrent infections, CMC, failure to thrive and autoimmunity could be listed as the warning signs of PID. A high presence of allergies was also reported in patients with associated autoimmunity and immunodeficiency [57]. The results of the present study evidenced a significant association of the S278R polymorphism of the *AIRE* gene with APECED-like conditions, including both patients affected by purely polyautoimmune disorders and patients affected by immune-dysregulatory manifestations/confirmed PID. This could be indicative of common molecular mechanisms that underlie the association of different autoimmune symptoms and even their association with immunodeficiency conditions. A trend of association was also observed with the IVS9+6 G>A polymorphism compared to the healthy controls (Figure 2B). Of note, the sera of two patients, Patients 11 and 34 in Table 1, tested positive for anti-IFNω Abs, known to be typical of the APECED syndrome. 

In light of the foregoing, we highlight the importance of analyzing known susceptibility genes in cohorts of patients. We corroborate the evidence that common *AIRE* polymorphisms may partially contribute to high complex polyautoimmunity phenotype predisposition in APECED-like patients. *AIRE* polymorphism identification may indeed act as a marker to emphasize the need to look for additional or novel genetic determinants playing in concert in causing polyautoimmunity and autoimmunity-immunodeficiency-associated conditions. Association studies based on the candidate gene approach and the recent advent of whole-exome sequencing will definitively help to elucidate the genetic risk factors responsible for these complex phenotypes. This will contribute to establishing an improved personalized diagnostic protocol and to ensure the development of targeted therapies in APECED-like conditions. 

## Figures and Tables

**Table 1:** Clinical and immunological characteristics of APECED-like patients.

Patient	Gender	Age at Referral (Years)	Diseases	Auto Abs	*AIRE* Gene Pattern *
1	M	5.42	Multiple allergies (5), bronchiectasis (5), asthmatic bronchitis (5), hyper IgE (5), alopecia (10), CMC (10)	TgAbs, TPOAbs, IAA, IA2Abs, TRGAbs, ASCA neg; **AMA**, **ASMA pos**	het. S278R
2	F	3.5	Cognitive retardation (1), CMC (3), arthralgia (3), congenital hypothyroidism (3), bronchiolitis (6), Behcet’s disease (11), very early onset inflammatory bowel disease (VEO-IBD)	TgAbs, TPOAbs, ANCA, ASCA, ENA, dsDNAAbs neg; **ANA pos**	het. S278R
3	M	6.19	Selective IgA deficiency (6), Crohn’s disease (6), arthralgia (7), T1D (9), pharmacological polyallergy (12)	IAA, IA2Abs, GADA, TRGAbs, anti-adrenal Abs, ANA, ANCA, dsDNAAbs neg	het. S278R het. R471C het. IVS9+6 G>A
4	M	3.7	Arthralgia (1), HT (4), recurrent fever episodes (4), selective IgA deficiency (4), vitiligo (5)	IAA, IA2Abs, GADA, TRGAbs, 21OHAbs, dsDNAAbs neg; **TgAbs**, **TPOAbs**, **ANA pos**	het. IVS9+6 G>A
5	F	5.91	Bronchopneumopathy (5), arthralgia (6), combined immunodeficiency (7), hepatopathy (7), mucociliary dyskinesia (7), eczematous dermatitis (11)	TgAbs, TPOAbs, ANCA, ASCA neg; **IAA**, **IA2Abs**, **GADA**, **ANA**, **dsDNAAbs pos**	het. IVS9+6 G>A
6	M	3.57	Atopic dermatitis (3), combined immunodeficiency (4), CMC (4)	TgAbs, TPOAbs, IAA, IA2Abs, GADA, TRGAbs, dsDNAAbs neg; **ANA**, **ENA**, **RoAbs**, **SCL70Abs**, **LaAbs**, **SMAbs**, **RNPAbs**, **JO1Abs pos**	het. S278R het. IVS9+6 G>A
7	F	1.83	CMC (1), pseudohypoparathyroidism (4), psychomotor retardation (4), coordination disorder (5), congenital onychopathy (12), pityriasis capitis (12), subclinical HT (12)	TgAbs, TPOAbs, ANCA, dsDNAAbs, PM/Scl 100 Abs neg; **ANA pos**	WT
8	M	7.83	Vitiligo (10), HT (10), inhalants allergy (10), selective IgA deficiency (12)	TRGAbs, anti-gliadin Abs, EMA, APCA, AMA, ANA, ANCA, ASCA, ENA, dsDNAAbs neg; **TgAbs**, **TPOAbs pos**	WT
9	M	4.29	Candidiasis, alopecia areata (4), subclinical HT (4) (familiarity for HT and T1D)	TgAbs, TPOAbs, IAA, IA2Abs, GADA, TRGAbs, APCA, ANA, ANCA, ENA neg	het. S278R
10	F	2.16	Childhood obesity (6), CMC (9), recurrent infections (9), T1D (9), preclinical hypoparathyroidism, het. MTHFR C677T homocysteine 9 mutation (9), leukopenia (10)	TgAbs, TPOAbs, anti-TSH-receptor Abs, TRGAbs, anti-gliadin Abs, ANA, anti-cardiolipin Abs, anti-beta2 glycoprotein Abs neg; **IAA**, **GADA pos**	WT
11	F	8.3	Tubulointerstitial nephritis (4), polyallergy (urticaria and food allergy) (9), chronic renal failure (10), frequent asthma episodes (9), bronchopneumopathy with pulmonary bronchiectasis, hypereosinophilia (9), chronic pancreatitis (10)	ANA, aPLAbs neg; **ANCA**, **ASCA**, **MPOAbs**, **anti-cardiolipin Abs pos**	WT
12	F	10.99	Alopecia (few months), HT (9), CMC, bronchitis, asthma, urinary tract infections, food allergies, failure to thrive, hypogammaglobulinemia, eczema, vitiligo, chronic gastritis, morphea	TgAbs, TPOAbs neg	WT
13	M	11.18	Addison’s disease, frequent infections, HT	Diabetes-related Abs, anti-gliadin Abs neg; **TgAbs**, **TPOAbs**, **21OHAbs pos**	het. S278R
14	M	11.18	CMC, HT, autoimmune pancytopenia	**TgAbs**, **TPOAbs pos**	WT
15	F	7.03	Alopecia (7), recurrent infections	TgAbs, TPOAbs, IAA, IA2Abs, GADA, anti-adrenal Abs, ENA, ASMA, ARA, APCA, ANCA, dsDNAAbs, LKMAbs, LC1Abs, anti-ribosome Abs neg; **ANA pos**	WT
16	F	10.51	HT (11), T1D (11), CMC	TPOAbs, IA2Abs, TRGAbs neg; **TgAbs**, **IAA**, **GADA pos**	WT
17	F	5.71	Turner syndrome, alopecia (3), HT, nail mycosis, cheilitis	**TgAbs**, **TPOAbs pos**	het. IVS9+6 G>A
18	M	2.8	Alopecia (3), recurrent respiratory infections, overweight (5)	TgAbs, TPOAbs, IAA, GADA, TRGAbs, APCA, anti-adrenal Abs, AMA, ANA, ARA, LC1Abs, LKMAbs, anti-ribosome Abs neg; **IA2Abs**, **ASMA**, **ENA pos**	WT
19	F		CMC, HT, autoimmune hypophysitis		WT
20	M	9.06	Alopecia (8), CVID (10), allergic rhinitis (10), onychodystrophy (12), palmar-plantar psoriasis (12), lactose intolerance (15) [54]	TgAbs, TPOAbs, IAA, IA2Abs, GADA, APCA, ANA, ASCA, ASMA, LKMAbs, dsDNAAbs, ENA, AMA, LC1Abs, anti-ribosome Abs, SCL70Abs neg	het. S250C
21	M	5.57	Hypoparathyroidism (12)	TgAbs, TPOAbs, IAA, IA2Abs, GADA, 21OHAbs neg	het. S278R het. IVS9+6 G>A
22	F	4.66	T1D (7)	TgAbs, IA2Abs, TRGAbs neg; **GADA pos**	het. S278R het. IVS9+6 G>A
23	M	2.31	T1D (2), linguistic retardation (2), celiac disease (21)	TgAbs, TPOAbs neg; **TRGAbs pos**	het. S278R het. IVS9+6 G>A
24	F	2.34	Congenital cataract, growth retardation (5), HT (5), autoimmune haemolytic anemia (7), juvenile idiopathic arthritis (9), A20 haploinsufficiency (16)	IAA, IA2Abs, APCA, anti-adrenal Abs, AMA, ANCA, ASMA, ARA, dsDNAAbs, LKMAbs, LC1Abs, anti-ribosome Abs neg; **TgAbs**, **TPOAbs**, **GADA**, **ANA**, **ENA pos**	het. S278R het. IVS9+6 G>A
25	F	3.72	T1D, HT (21), vitiligo (21)	Anti-TSH-receptor Abs, IA2Abs, GADA, TRGAbs, anti-adrenal Abs neg; **TgAbs**, **TPOAbs**, **IAA pos**	het. S278R het. IVS9+6 G>A
26	F	4.81	T1D, Down’s syndrome, Basedow’s disease (32)	TgAbs, TPOAbs, TRGAbs, APCA, anti-adrenal Abs, AMA, ARA, LKMAbs, LC1Abs, anti-ribosome Abs neg; **ASMA pos**	het. S278R
27	F	11.25	HT, celiac disease	IAA, IA2Abs, GADA, TRGAbs neg; **TgAbs**, **TPOAbs pos**	het. S278R
28	F	12.43	Alopecia areata (5), parapsoriasis (12), HT (12), mycosis fungoides (13)		het. S278R
29	M	8.41	Isolated hypoparathyroidism (8)	TgAbs, TPOAbs, TRGAbs, anti-adrenal Abs neg	het. S278R
30	F	5.78	Alopecia (6), onychodystrophy (13)	TgAbs, TPOAbs, IAA, IA2Abs, TRGAbs, APCA, anti-adrenal Abs, AMA, ASMA, ARA, LKMAbs, LC1Abs, anti-ribosome Abs neg; **GADA**, **ANA pos**	het. S278R
31	F	10.91	Primary hypoparathyroidism		het. S278R
32	M	10.08	Hypoparathyroidism		het. S278R
33	F		Addison’s disease, vitiligo		het. S278R het. IVS9+6 G>A
34	F	14.35	Central hypoadrenalism (15), HT (15), bronchiectasis (15), CIDP (21)	IAA, GADA, ANA, ANCA neg; **TgAbs**, **TPOAbs pos**	WT
35	F	1	Early menarche from ovarian cyst (9), alopecia (16), HT (17), subclinical hypoadrenalism (17)	TgAbs, TRGAbs, APCA, anti-adrenal Abs, AMA, ASMA, ARA, LKMAbs, LC1Abs, SLA/LPAbs, Sp100Abs, gp210Abs, anti-cardiolipin Abs neg; **TPOAbs**, **ANA pos**	het. S278R
36	F		Hypoparathyroidism, Addison’s disease, secondary ovarian failure		WT
37	F	1	Alopecia (3), HT (7)	TgAbs, IAA, IA2Abs, TRGAbs, APCA, anti-adrenal Abs, AMA, ASMA, ARA, LKMAbs, LC1Abs, anti-ribosome Abs, anti-cardiolipin Abs neg; **TPOAbs**, **GADA**, **ANA pos**	het. S278R
38	F	6.15	Alopecia (1), nail dystrophy, HT (5), allergic rhinitis (11), arthralgia (11), recurrent infections in pediatric age	IAA, GADA, TRGAbs, APCA, ANCA, ASMA, ARA, dsDNAAbs, LKMAbs, anti-ribosome Abs neg; **TgAbs**, **TPOAbs**, **IA2Abs**, **ANA pos**	het. IVS9+6 G>A
39	M	3.84	T1D, GH deficit, HT, autoimmune leukopenia	TRGAbs, anti-adrenal Abs neg; **TgAbs**, **TPOAbs pos**	het. IVS9+6 G>A
40	F	15.42	Addison’s disease, HT, celiac disease (4)	TgAbs, TPOAbs, IAA, IA2Abs, GADA, APCA, ANA, AMA, ASMA, LKMAbs, ARA, LC1Abs, anti-ribosome Abs neg; **TRGAbs**, **anti-adrenal Abs pos**	WT
41	M		alopecia, HT, celiac disease	IAA, IA2Abs, GADA, anti-adrenal Abs, dsDNAAbs, aPLAbs, anti-cardiolipin Abs neg; **TgAbs**, **TPOAbs**, **TRGAbs**, **ANA**, **ANCA pos**	het. S278R
42	F		Addison’s disease, HT, psoriasis		WT
43	F	7.97	Hypoadrenalism (8), hypothyroidism (10)	IAA, IA2Abs, GADA, TRGAbs, AMA, ASMA, ARA, LKMAbs, LC1Abs, anti-ribosome Abs neg; **TgAbs**, **TPOAbs**, **APCA, anti-adrenal Abs pos**	WT
44	F	5.64	Celiac disease (5), T1D (7), HT (7)	TgAbs, TRGAbs neg; **TPOAbs**, **IAA**, **IA2Abs**, **GADA pos**	WT
45	F	5.19	T1D (4), autoimmune hepatitis (5), HT (5)	TPOAbs, TRGAbs, APCA, ANA, AMA, ASMA, ARA, ANCA, LKMAbs, LC1Abs, anti-ribosome Abs neg; **TgAbs pos**	WT
46	F	1.8	T1D (2), celiac disease (3), autoimmune hepatitis (13)	TgAbs, TPOAbs, TRGAbs, APCA, ANCA, ANA, AMA, ASMA, ARA, LKMAbs, LC1Abs, anti-ribosome Abs neg; **IAA**, **IA2Abs**, **GADA pos**	WT
47	F	11.71	Arthralgia (4), psoriasis (6), HT (9), T1D (9), gastritis (12)	TgAbs, TRGAbs, APCA, anti-adrenal Abs, dsDNAAbs, AMA, ASMA, ARA, ASCA, LKMAbs, LC1Abs, anti-ribosome Abs neg; **TPOAbs**, **ANA**, **ANCA pos**	WT
48	F	14.77	Addison’s disease, MERS	IAA, IA2Abs, GADA, APCA, ANA, ENA, AMA, ASMA, ARA, LKMAbs, LC1Abs, aPLAbs, anti-cardiolipin Abs, anti-ribosome Abs, anti-beta2 microglobulin Abs neg; **TRGAbs**, **anti-adrenal Abs pos**	het. S278R

* Mutations and polymorphisms; WT: Wild Type; het: heterozygous; NT: not tested; neg: negative; pos: positive; GH: growth hormone; MERS: mild encephalitis/encephalopathy with reversible splenial lesion. 21OHAbs: 21OH hydroxylase Abs; JO1Abs: histidyl-tRNA synthetase Abs; SCL70Abs: topoisomerase I Abs; SSA/Ro Abs: anti-Sjögren’s-syndrome-related antigen A Abs; SSB/LA Abs: anti-Sjögren’s-syndrome-related antigen B Abs; SMAbs: anti-Smith Abs; PM/Scl 100 Abs: polymyositis (PM)/Scl-100 Abs; RNPAbs: anti-U1 ribonucleoprotein Abs; ASCA: anti-Saccharomyces cerevisiae Abs; MPOAbs: anti-myeloperoxidase Abs; LC1Abs: liver cytosol Type 1 Abs; aPLAbs: anti-phospholipids Abs; SLA/LPAbs: antibodies against soluble liver antigen/liver-pancreas; Sp100Abs: anti-Sp100 Abs; gp210Abs: anti-glycoprotein-210 Abs. SNPs: single-nucleotide polymorphisms. In brackets, age of disease onset of symptoms is shown.

## Data Availability

The original contributions presented in the study are included in the article. Further inquiries can be directed to the corresponding author.

## References

[1] Fierabracci A., Milillo A., Locatelli F., Fruci D. (2012). The putative role of endoplasmic reticulum aminopeptidases in autoimmunity: Insights from genomic-wide association studies. Autoimmun. Rev..

[2] Amaya-Uribe L., Rojas M., Azizi G., Anaya J.M., Gershwin M.E. (2019). Primary immunodeficiency and autoimmunity: A comprehensive review. J. Autoimmun..

[3] Husebye E.S., Anderson M.S., Kämpe O. (2018). Autoimmune Polyendocrine Syndromes. N. Engl. J. Med..

[4] Betterle C., Sabbadin C., Scaroni C., Presotto F., Colao A.M., Jaffrain-Rea M.L., Beckers A. (2019). Autoimmune polyendocrine syndromes (APS) or multiple autoimmune syndromes (MAS) an overview. Polyendocrine Disorders and Endocrine Neoplastic Syndromes.

[5] Schmidt R.E., Grimbacher B., Witte T. (2017). Autoimmunity and primary immunodeficiency: Two sides of the same coin?. Nat. Rev. Rheumatol..

[6] Walter E.J., Ayala I.A., Milojevic D. (2019). Autoimmunity as a continuum in primary immunodeficiency. Curr. Opin. Pediatr..

[7] Reits E., Neefjes J. (2022). HLA molecules in transplantation, autoimmunity and infection control: A comic book adventure. HLA.

[8] Tobón G.J., Pers J.O., Cañas C.A., Rojas-Villarraga A., Youinou P., Anaya J.M. (2012). Are autoimmune diseases predictable?. Autoimmun. Rev..

[9] Hosseini A., Gharibi T., Marofi F., Babaloo Z., Baradaran B. (2020). CTLA-4: From mechanism to autoimmune therapy. Int. Immunopharmacol..

[10] Gough S.C., Walker L.S., Sansom D.M. (2005). CTLA4 gene polymorphism and autoimmunity. Immunol. Rev..

[11] Kailashiya V., Sharma H.B., Kailashiya J. (2019). Role of CTLA4 A49G polymorphism in systemic lupus erythematosus and its geographical distribution. J. Clin. Pathol..

[12] Wing J.B., Tanaka A., Sakaguchi S. (2019). Human FOXP3+ Regulatory T Cell Heterogeneity and Function in Autoimmunity and Cancer. Immunity.

[13] Mailer R.K., Mailer R.K. (2020). IPEX as a Consequence of Alternatively Spliced FOXP3. Front. Pediatr..

[14] Dikiy S., Li J., Bai L., Jiang M., Janke L., Zong X., Hao X., Hoyos B., Wang Z.M., Xu B. (2021). A distal Foxp3 enhancer enables interleukin-2 dependent thymic Treg cell lineage commitment for robust immune tolerance. Immunity.

[15] Durães C., Moreira C.S., Alvelos I., Mendes A., Santos L.R., Machado J.C., Melo M., Esteves C., Neves C., Sobrinho-Simões M. (2014). Polymorphisms in the TNFA and IL6 genes represent risk factors for autoimmune thyroid disease. PLoS ONE.

[16] Abd El-Raheem T., Mahmoud R.H., Hefzy E.M., Masoud M., Ismail R., Aboraia N.M.M. (2020). Tumor necrosis factor (TNF)-α- 308 G/A gene polymorphism (rs1800629) in Egyptian patients with alopecia areata and vitiligo, a laboratory and in silico analysis. PLoS ONE.

[17] Gianchecchi E., Palombi M., Fierabracci A. (2013). The putative role of the C1858T polymorphism of protein tyrosine phosphatase PTPN22 gene in autoimmunity. Autoimmun. Rev..

[18] Vang T., Congia M., Macis M.D., Musumeci L., Orrú V., Zavattari P., Nika K., Tautz L., Taskén K., Cucca F. (2005). Autoimmune-associated lymphoid tyrosine phosphatase is a gain-of-function variant. Nat. Genet..

[19] Fierabracci A., Pellegrino M., Frasca F., Kilic S.S., Betterle C. (2018). APECED in Turkey: A case report and insights on genetic and phenotypic variability. Clin. Immunol..

[20] Fierabracci A. (2011). Recent insights into the role and molecular mechanisms of the autoimmune regulator (AIRE) gene in autoimmunity. Autoimmun. Rev..

[21] Garelli S., Dalla Costa M., Sabbadin C., Barollo S., Rubin B., Scarpa R., Masiero S., Fierabracci A., Bizzarri C., Crinò A. (2021). Autoimmune polyendocrine syndrome type 1: An Italian survey on 158 patients. J. Endocrinol. Investig..

[22] Kekäläinen E., Tuovinen H., Joensuu J., Gylling M., Franssila R., Pöntynen N., Talvensaari K., Perheentupa J., Miettinen A., Arstila T.P. (2007). A defect of regulatory T cells in patients with autoimmune polyendocrinopathy-candidiasis-ectodermal dystrophy. J. Immunol..

[23] Fierabracci A., Arena A., Toto F., Gallo N., Puel A., Migaud M., Kumar M., Chengappa K.G., Gulati R., Negi V.S. (2021). Autoimmune polyendocrine syndrome type 1 (APECED) in the Indian population: Case report and review of a series of 45 patients. J. Endocrinol. Investig..

[24] Larosa M.D.P., Mackenzie R., Burne P., Garelli S., Barollo S., Masiero S., Rubin B., Chen S., Furmaniak J., Betterle C. (2017). Assessment of autoantibodies to interferon-ω in patients with autoimmune polyendocrine syndrome type 1: Using a new immunoprecipitation assay. Clin. Chem. Lab. Med..

[25] Azizi G., Yazdani R., Rae W., Abolhassani H., Rojas M., Aghamohammadi A., Anaya J.M. (2018). Monogenic polyautoimmunity in primary immunodeficiency diseases. Autoimmun. Rev..

[26] Fierabracci A. (2011). The role of heterozygous mutations of the autoimmune regulator gene (AIRE) in non-APECED autoimmunity: A comment on recent findings. Clin. Endocrinol..

[27] Anderson G., Takahama Y. (2012). Thymic epithelial cells: Working class heroes for T cell development and repertoire selection. Trends Immunol..

[28] Sedivá A., Ciháková D., Lebl J. (2002). Immunological findings in patients with autoimmune polyendocrinopathy-candidiasis-ectodermal dystrophy (APECED) and their family members: Are heterozygotes subclinically affected?. J. Pediatr. Endocrinol. Metab..

[29] Cervato S., Morlin L., Albergoni M.P., Masiero S., Greggio N., Meossi C., Chen S., Larosa M.D.P., Furmaniak J., Smith B.R. (2010). AIRE gene mutations and autoantibodies to interferon omega in patients with chronic hypoparathyroidism without APECED. Clin. Endocrinol..

[30] Tóth B., Wolff A.B.S., Halász Z., Tar A., Szüts P., Ilyés I., Erdos M., Szegedi G., Husebye E.S., Zeher M. (2010). Novel sequence variation of AIRE and detection of interferon-omega antibodies in early infancy. Clin. Endocrinol..

[31] Alimohammadi M., Björklund P., Hallgren A., Pöntynen N., Szinnai G., Shikama N., Keller M.P., Ekwall O., Kinkel S.A., Husebye E.S. (2008). Autoimmune polyendocrine syndrome type 1 and NALP5, a parathyroid autoantigen. N. Engl. J. Med..

[32] Oftedal B.E., Wolff A.S., Bratland E., Kämpe O., Perheentupa J., Myhre A.G., Meager A., Purushothaman R., Ten S., Husebye E.S. (2008). Radioimmunoassay for autoantibodies against interferon omega; its use in the diagnosis of autoimmune polyendocrine syndrome type I. Clin. Immunol..

[33] Gossard A.A., Lindor K.D. (2012). Autoimmune hepatitis: A review. J. Gastroenterol..

[34] Vogel A., Liermann H., Harms A., Strassburg C.P., Manns M.P., Obermayer-Straub P. (2001). Autoimmune regulator AIRE: Evidence for genetic differences between autoimmune hepatitis and hepatitis as part of the autoimmune polyglandular syndrome type 1. Hepatology.

[35] Lankisch T.O., Strassburg C.P., Debray D., Manns M.P., Jacquemin E. (2005). Detection of autoimmune regulator gene mutations in children with type 2 autoimmune hepatitis and extrahepatic immune-mediated diseases. J. Pediatr..

[36] Lankisch T.O., Mourier O., Sokal E.M., Habes D., Lacaille F., Bridoux-Henno L., Hermeziu B., Lenaerts C., Strassburg C.P., Jacquemin E. (2009). AIRE gene analysis in children with autoimmune hepatitis type I or II. J. Pediatr. Gastroenterol. Nutr..

[37] Tazi-Ahnini R., Cork M.J., Gawkrodger D.J., Birch M.P., Wengraf D., McDonagh A.J., Messenger A.G. (2002). Role of the autoimmune regulator (AIRE) gene in alopecia areata: Strong association of a potentially functional AIRE polymorphism with alopecia universalis. Tissue Antigens.

[38] Scott H.S., Heino M., Peterson P., Mittaz L., Lalioti M.D., Betterle C., Cohen A., Seri M., Lerone M., Romeo G. (1998). Common mutations in autoimmune polyendocrinopathy-candidiasis-ectodermal dystrophy patients of different origins. Mol. Endocrinol..

[39] Faiyaz-Ul-Haque M., Bin-Abbas B., Al-Abdullatif A., Abdullah Abalkhail H., Toulimat M., Al-Gazlan S., Almutawa A.M., Al-Sagheir A., Peltekova I., Al-Dayel F. (2009). Novel and recurrent mutations in the AIRE gene of autoimmune polyendocrinopathy syndrome type 1 (APS1) patients. Clin. Genet..

[40] Pforr J., Blaumeiser B., Becker T., Freudenberg-Hua Y., Hanneken S., Eigelshoven S., Cuyt I., De Weert J., Lambert J., Kruse R. (2006). Investigation of the p.Ser278Arg polymorphism of the autoimmune regulator (AIRE) gene in alopecia areata. Tissue Antigens..

[41] Ferrera F., Rizzi M., Sprecacenere B., Balestra P., Sessarego M., Di Carlo A., Filaci G., Gabrielli A., Ravazzolo R., Indiveri F. (2007). AIRE gene polymorphisms in systemic sclerosis associated with autoimmune thyroiditis. Clin. Immunol..

[42] Vaidya B., Imrie H., Geatch D.R., Perros P., Ball S.G., Baylis P.H., Carr D., Hurel S.J., James R.A., Kelly W.F. (2000). Association analysis of the cytotoxic T lymphocyte antigen-4 (CTLA-4) and autoimmune regulator-1 (AIRE-1) genes in sporadic autoimmune Addison’s disease. J. Clin. Endocrinol. Metab..

[43] Nithiyananthan R., Heward J.M., Allahabadia A., Barnett A.H., Franklyn J.A., Gough S.C. (2000). A heterozygous deletion of the autoimmune regulator (AIRE1) gene, autoimmune thyroid disease, and type 1 diabetes: No evidence for association. J. Clin. Endocrinol. Metab..

[44] Török H.P., Tonenchi L., Glas J., Schiemann U., Folwaczny C. (2004). No significant association between mutations in exons 6 and 8 of the autoimmune regulator (AIRE) gene and inflammatory bowel disease. Eur. J. Immunogenet..

[45] Turunen J.A., Wessman M., Forsblom C., Kilpikari R., Parkkonen M., Pöntynen N., Ilmarinen T., Ulmanen I., Peltonen L., Groop P.H. (2006). Association analysis of the AIRE and insulin genes in Finnish type 1 diabetic patients. Immunogenetics.

[46] Palma A., Gianchecchi E., Palombi M., Luciano R., Di Carlo P., Crinò A., Cappa M., Fierabracci A. (2013). Analysis of the autoimmune regulator gene in patients with autoimmune non-APECED polyendocrinopathie. Genomics.

[47] Oftedal B.E., Hellesen A., Erichsen M.M., Bratland E., Vardi A., Perheentupa J., Kemp E.H., Fiskerstrand T., Viken M.K., Weetman A.P. (2015). Dominant Mutations in the Autoimmune Regulator AIRE Are Associated with Common Organ-Specific Autoimmune Diseases. Immunity.

[48] Cetani F., Barbesino G., Borsari S., Pardi E., Cianferotti A., Pinchera C., Marcocci F. (2001). A novel mutation of the autoimmune regulator gene in an Italian kindred with autoimmune polyendocrinopathy-candidiasis-ectodermal dystrophy, acting in a dominant fashion and strongly cosegregating with hypothyroid autoimmune thyroiditis. J. Clin. Endocrinol. Metab..

[49] Colobran R., Giménez-Barcons M., Marín-Sánchez A., Porta-Pardo E., Pujol-Borrell R. (2016). AIRE genetic variants and predisposition to polygenic autoimmune disease: The case of Graves’ disease and a systematic literature review. Hum. Immunol..

[50] Laisk T., Lepamets M., Koel M., Abner E., Mägi R., Estonian Biobank Research Team (2021). Genome-wide association study identifies five risk loci for pernicious anemia. Nat. Commun..

[51] Eriksson D., Røyrvik E.C., Aranda-Guillén M., Berger A.H., Landegren N., Artaza H., Hallgren A., Grytaas M.A., Ström S., Bratland E. (2021). GWAS for autoimmune Addison’s disease identifies multiple risk loci and highlights AIRE in disease susceptibility. Nat. Commun..

[52] Fierabracci A. (2016). Type 1 Diabetes in Autoimmune Polyendocrinopathy-Candidiasis-Ectodermal Dystrophy Syndrome (APECED): A “Rare” Manifestation in a “Rare” Disease. Int. J. Mol. Sci..

[53] Odineal D.D., Gershwin M.E. (2020). The Epidemiology and Clinical Manifestations of Autoimmunity in Selective IgA Deficiency. Clin. Rev. Allergy Immunol..

[54] Bellacchio E., Palma A., Corrente S., Di Girolamo F., Kemp H.E., Di Matteo G., Comelli L., Carsetti R., Cascioli S., Cancrini C. (2014). The possible implication of the S250C variant of the autoimmune regulator protein in a patient with autoimmunity and immunodeficiency: In silico analysis suggests a molecular pathogenic mechanism for the variant. Gene.

[55] Picard C., Gaspar H.B., Al-Herz W., Bousfiha A., Casanova J.L., Chatila T., Crow Y.J., Cunningham-Rundles C., Etzioni A., Franco J.L. (2018). International Union of Immunological Societies: 2017 Primary Immunodeficiency Diseases Committee Report on Inborn Errors of Immunity. J. Clin. Immunol..

[56] Tangye S.G., Al-Herz W., Aziz Bousfiha A., Cunningham-Rundles C., Franco J.L., Holland S.M., Klein C., Morio T., Oksenhendler E., Picard C. (2021). The Ever-Increasing Array of Novel Inborn Errors of Immunity: An Interim Update by the IUIS Committee. J. Clin. Immunol..

[57] Ludvigsson J.F., Neovius M., Hammarström L. (2014). Association between IgA deficiency & other autoimmune conditions: A population-based matched cohort study. J. Clin. Immunol..

